# Evaluation of vaccine-induced antibody responses against SARS-CoV-2 in multiple myeloma patients from the northeastern region of Mexico

**DOI:** 10.1016/j.htct.2026.106448

**Published:** 2026-04-06

**Authors:** Rosario Salazar-Riojas, Dalila M. Alvarado-Navarro, María L. Ruiz-de la Cruz, Lorena Nefertiti Castro-Fuentes, Karina E. Vazquez-Hernandez, David Gomez-Almaguer, Adrian G. Rosas-Taraco

**Affiliations:** a Universidad Autonoma de Nuevo Leon, Facultad de Medicina y Hospital Universitario “Dr. Jose E. Gonzalez”, Service of Hematology. Monterrey, 64720, Mexico; b Universidad Autonoma de Nuevo Leon, Facultad de Medicina y Hospital Universitario “Dr. Jose E. Gonzalez”, Service and Department of Immunology. Monterrey, N.L. 64460, Mexico

**Keywords:** SARS-CoV-2 vaccines, Multiple myeloma, Antibodies, Vaccine efficacy, Humoral immunity, Subclinical COVID-19 infection

## Abstract

**Objective:**

To evaluate immunoglobulin G antibody responses against Severe Acute Respiratory Syndrome Coronavirus 2 in multiple myeloma patients in the northeastern region of Mexico following different COVID-19 vaccination regimens.

**Methods:**

This retrospective study included 33 multiple myeloma patients, and 28 healthy controls vaccinated with mRNA (BNT162b2, mRNA-1273) or adenovector (AZD1222) vaccines. Anti-spike receptor-binding domain (RBD) and anti-nucleocapsid immunoglobulin G levels were measured. Laboratory parameters, including monocyte and lymphocyte counts and serum-free light chains, were analyzed. Statistical comparisons used ANOVA, Kruskal-Wallis, and logistic regression.

**Results:**

No significant differences in anti-spike receptor-binding domain immunoglobulin G levels were observed between multiple myeloma patients and healthy controls after one vaccine dose. Multiple myeloma patients who received ≥3 doses showed higher anti-spike receptor-binding domain immunoglobulin G levels compared to single-dose recipients. Anti-nucleocapsid antibodies (indicative of prior subclinical infection) were detected in 51.5% of multiple myeloma patients and correlated with elevated anti-spike receptor-binding domain levels. Patients in the third anti-spike immunoglobulin G quartile (10,427.9–23,954.0 AU/mL) had higher monocyte counts compared with patients in the lower quartiles. No correlations were found between anti-spike immunoglobulin G and lymphocyte counts, serum proteins, or gamma globulins. Abnormal kappa/lambda light chain ratios did not impair antibody responses in nucleocapsid-positive patients.

**Conclusion:**

Multiple myeloma patients achieved comparable immunoglobulin G responses to healthy controls after one vaccine dose, with enhanced responses following ≥3 doses. Subclinical infections may augment humoral immunity. Elevated monocyte counts are indicative of innate immune activation following the administration of the vaccine. These findings support prioritizing booster doses for multiple myeloma patients despite immunosuppressive therapies.

## Introduction

Multiple myeloma (MM) is a hematological malignancy characterized by the clonal expansion of plasma B cells that are committed to producing a specific type of immunoglobulin, which can serve as a tumor antigen. The disease is also marked by a heterogeneous accumulation of tumor cells within the bone marrow, leading to clinical complications such as anemia, lytic bone lesions, hypercalcemia, renal failure, and increased susceptibility to infections. This heightened vulnerability to infections is directly associated with the aberrant expression of transcription factors and the impaired co-stimulatory signaling between B cells and T helper cells during antigen presentation [1].

The immunocompromised state in MM patients, further exacerbated by immunomodulatory treatments and, more recently, by using anti-CD38 monoclonal antibodies, can significantly impair adaptive immunity and increase the risk of infections. During the COVID-19 pandemic, vaccination was strongly recommended for all hematological patients, as they are a high-risk group for Severe Acute Respiratory Syndrome Coronavirus 2 (SARS-CoV-2) infection and develop severe disease [2]. Although some MM patients may exhibit reduced responses to vaccines, vaccination still provides potential benefits in this vulnerable population [3]. Immunosuppression in MM patients affects all effector cells of the immune system [1,4]. Given this immunological landscape, which impairs effective antigen presentation and hinders the development of adaptive immunity and long-term protection against infections, it is crucial to assess the long-term efficacy of vaccines in these patients to establish the optimal vaccination schedule.

Immunological traits, which are associated with a low specific antibody response to vaccination, are commonly found in MM patients. These traits include older age, kidney function alteration, lymphocytopenia, low immunoglobulin levels, and the need for a second line of treatment, and also in patients not in complete remission [5,6].

Therefore, this study aims to evaluate the anti-spike receptor-binding domain (RBD) immunoglobulin G (IgG) response in MM patients who followed different vaccination regimens during the 2020 COVID-19 pandemic. The patients were attended at the University Hospital in the Northeast of Mexico.

## Patients and methods

### Study group

Patients with confirmed diagnosis of MM and who were being treated in the University Hospital were invited to participate. Patients previously vaccinated against SARS-CoV2 with mRNA vaccines (BNT162b2 and mRNA1273) or the adenovector vaccine AZD1222 vaccine were eligible if they were aged 18 to 65 and had an Eastern Cooperative Oncology (ECOG) performance status score between 0 and 2 (on a 5-point scale, where higher scores represent increased disability). Individuals were excluded if they had active disease, central nervous system involvement, primary amyloidosis, or showed inadequate hematologic, hepatic, renal, or cardiac function. The enrolled patients and controls should have at least received a single dose of one of the following three COVID-19 vaccines authorized for emergency or full use during the study period (2020–2021): the mRNA vaccines BNT162b2 (Pfizer-BioNTech) and mRNA-1273 (Moderna), and the adenoviral vector vaccine AZD1222 (AstraZeneca-Oxford).

Healthy controls were recruited through convenience sampling from hospital staff and patient companions. The following exclusion criteria were applied specifically to the control group: immunosuppressive conditions (including autoimmune diseases, immunomodulator use, or HIV infection), active infection at recruitment, history of hematologic malignancies, and prior organ or stem cell transplantation. All controls received their SARS-CoV-2 vaccinations according to the national guidelines during the same epidemiological period as patients, ensuring comparable exposure to circulating variants.

Patients and controls provided both written and verbal informed consent for trial participation. Patient clinicodemographic characteristics and outcomes were retrospectively gathered from their electronic medical records. No interventions were conducted as part of this study. This research adhered to institutional guidelines and received approval from the Institutional Review Board (IN23–00,003) and the School of Medicine Ethics Committee. Additionally, the study followed Good Clinical Practice standards Practices.

### Assessment of serological response

This study included 33 patients with MM and 28 healthy controls. Blood samples were obtained from each patient and control; the sera were recovered by centrifugation and stored at −80 °C until use. Total IgG antibodies directed against the RBD of the SARS-CoV-2 spike protein were quantified using the SARS-CoV-2 IgG II Quant assay (Abbott). Additionally, qualitative detection of IgG directed against the nucleocapsid protein was performed using the SARS-CoV-2 IgG assay (Abbott), in accordance with the manufacturer's established protocols. The cutoff for RBD IgG antibodies was ≥250 AU/mL according to previous reports [5] and the cutoff for the nucleocapsid antibodies index was ≥1.40 S/C, according to the manufacturer’s guidelines.

### Laboratory findings

A complete blood count and routine serological assessment of MM patients, including total proteins, gamma globulin, and serum-free chains, were performed along with the serum sample for IgG antibody determination with the results being obtained from electronic health records following the attainment of informed consent from all participants and formal approval from the Institutional Review Board.

### Statistical analysis

Statistical analyses were performed using Graph Pad Prism-8 (La Jolla, GraphPad Software). Normality was analyzed using Shapiro-Wilk test. The differences between the group means were evaluated using one-way ANOVA followed by Tukey's post hoc test, or Kruskal-Wallis followed by Dunn's post hoc test for multiple comparisons. P-values <0.05 was considered statistically significant.

## Results

A total of 33 patients and 28 healthy controls were included in the study. The patient group comprised 20 males (60.6 %) with a mean age of 65.76 ± 11.2 years, while the healthy control group consisted of nine males (32.1 %) with a mean age of 47 ± 22.0 years.

### Patient characteristics

Patients were treated with bortezomib, lenalidomide and dexamethasone (VRD) induction therapy. Nineteen patients (57.6 %) underwent autologous stem cell transplant (ASCT) with the antibody levels being obtained at least six months post-transplant. Maintenance treatment included lenalidomide 10 mg/day or bortezomib 1.3 mg/m^2^ every other week. All patients were in very good partial response or better at the time of study.

The evaluation of serum immunoglobulins and light chains demonstrated a notable predominance of IgG, accounting for 71.4 % (95 % confidence interval [95 % CI]: 52.9–84.9 %) of the cases analyzed. Remarkably, even at the lower limit of the confidence interval, IgG constituted the most prevalent isotype, appearing in more than 50 % of the cases. In the analysis of light chains, kappa was observed to have a slightly higher prevalence, represented in 53.6 % (95 % CI: 35.8–70.5 %), compared to lambda, which accounted for 46.4 %. However, this difference lacked statistical significance.

A distinct trend was evident in the immunoglobulin A (IgA) cases, where lambda light chains observed in 75 % of these cases (6 out of 8), predominated. Nonetheless, the limited sample size of IgA patients led to a considerable degree of uncertainty in this estimate (95 % CI:10.0–40.1 %).

Patient characteristics, vaccination status, anti-SARS-CoV-2 antibody levels, and laboratory findings are summarized in Table 1. Notably, only one patient tested negative for IgG spike-RBD antibodies, with a 2.2 level of AU/mL. Additionally, 17 patients (51.5 %) were positive for IgG nucleocapsid antibodies. Regarding vaccination status, 13 patients (39.4 %) had received a single vaccine dose. Of these, 12 (92.3 %) were vaccinated with BNT162b2, while only one (7.7 %) received AZD1222. The only patient who was negative for spike RBD antibodies received one dose of the BNT162b2 vaccine. In respect the administration of multiple doses of the vaccine, no patients reported the use of two doses. Twelve patients (36.4 %) reported they had received three doses, consisting of various combinations of BNT162b2 (B), AZD1222 (A), and mRNA-1273 (M). Of these, six (50 %) received three doses of AZD1222, two (16.7 %) received the BBA combination, and the remaining patients received the following regimens: BAA (8.3 %), ABB (8.3 %), BBB (8.3 %), and MMM (8.3 %). Seven patients (21.2 %) had received four doses of the vaccine: six (85.7 %) received four doses of AZD1222, and one patient (14.3 %) received four doses of mRNA-1273. Finally, one patient had received five doses, with the vaccination scheme being BBMMB.

**Table 1:** Descriptive statistics for the study population.

Total patients	33
Males - n ( %)	20 (60.6)
Age (years) - mean (SD)	65.76 (11.2)
**Vaccination status** (*n* = 33)	
Total number of vaccines received - median (IQR)	3 (1–3.5)
Time elapsed since last vaccination - n ( %)	
<1 year	5 (15.1)
>1 year	18 (54.5)
Not reported/ Does not remember	10 (30.3)
Brand of the last dose received - n ( %)	
Pfizer (BNT162b2)	22 (66.6)
AstraZeneca (AZD1222)	9 (27.3)
Moderna (mRNA-1273)	2 (6.1)
Modality of the last dose received - n ( %)	
mRNA	24 (72.7)
Adenovector	9 (27.3)
**Anti SARS-CoV2 antibodies**	
IgG anti-spike-RBD (AU/mL) - median (IQR)	10,428 (2323–26,048)
IgG anti-spike response - n ( %)	
Positive	32 (96.97)
Negative	1 (3.03)
IgG anti-nucleocapsid (S/C index) - median (IQR)	1.71 (0.08–5.68)
IgG anti-nucleocapsid response - n ( %)	
Positive	17 (51.5)
Negative	16 (48.5)
**Laboratory findings**	
Hemoglobin (g/dL) (*n* = 32) - mean (SD)	12.66 (1.80)
White blood cell count (x10^3^/µL), (*n* = 32) - mean (SD)	5.56 (1.98)
Platelets (x10^3^/µL) (*n* = 32) - median (IQR)	191.00 (151.0–261.8)
Lymphocytes (x10^3^/µL) (*n* = 32) - mean (SD)	1.71 (0.71)
Monocytes (x10^3^/µL) (*n* = 32) - median (IQR)	0.61 (0.422–0.80)
Neutrophils (x10^3^/µL) (*n* = 32) - mean (SD)	2.78 (1.21)
Eosinophils (x10^3^/µL) (*n* = 32) - median (IQR)	0.15 (0.09–0.22)
Basophils (x10^3^/µL) (*n* = 32) - median (IQR)	0.02 (0.01–0.04)
Serum total proteins (g/dL) (*n* = 32) - mean (SD)	6.50 (0.83)
Serum gamma globulin (g/dL) (*n* = 31) - median (IQR)	0.80 (0.40–1.20)
Serum kappa free light chains (mg/L) (*n* = 27) - median (IQR)	31.27 (15.44–68.08)
Serum lambda free light chains (mg/L) (*n* = 27) - median (IQR)	21.55 (13.58–35.70)
Serum kappa/lambda ratio (*n* = 27) - median (IQR)	1.36 (0.84–4.12)
**Characteristics of controls**	
Total (n)	28
Males - n ( %)	9 (32.1)
Age (years) - mean (SD)	47 (22.0)
**Brand of the last dose received - n (%)**	
Pfizer (BNT162b2)	18 (64.3)
Moderna (mRNA-1273)	10 (35.7)
**Modality of the last dose received n (%)**	
mRNA	28 (100)
**Anti SARS-CoV2 antibodies**	
IgG anti-spike-RBD (AU/mL) - median (IQR)	8201 (1354–19,219)
IgG anti-spike response - n ( %)	
Positive	26 (92.9)
Negative	2 (7.1)

mRNA: Messenger Ribonucleic Acid; Adenovector: Adenovirus vector; IgG: Immunoglobulin G;.

*Data is presented as mean ± standard deviation (SD) for normally distributed variables and as median (interquartile range, IQR) for non-normally distributed variables.

### Control characteristics

A total of 28 healthy controls were included for the assessment of antibody response after a single dose of the mRNA vaccines BNT162b2 and mRNA1273. The healthy control group consisted of 28 participants, with nine males (32.1 %) and a mean age of 47 years (standard deviation ± 22.0). All controls received mRNA vaccines as their most recent dose, with Pfizer-BioNTech (BNT162b2) being the most common (64.3 %; *n* = 18), followed by Moderna (mRNA-1273 - 35.7 %; *n* = 10). Serological analysis revealed robust immunogenicity, with 92.9 % (*n* = 26) showing positive anti-spike-RBD IgG responses. The median antibody titer was 8201 AU/mL (interquartile range [IQR]: 1354–19,219), indicating substantial variation in individual humoral responses. Only two participants (7.1 %) had negative antibody results, potentially reflecting individual differences in immune reactivity to vaccination.

### Serum immunoglobulin G response to the severe acute respiratory syndrome coronavirus 2 vaccination

**Figure 1:**
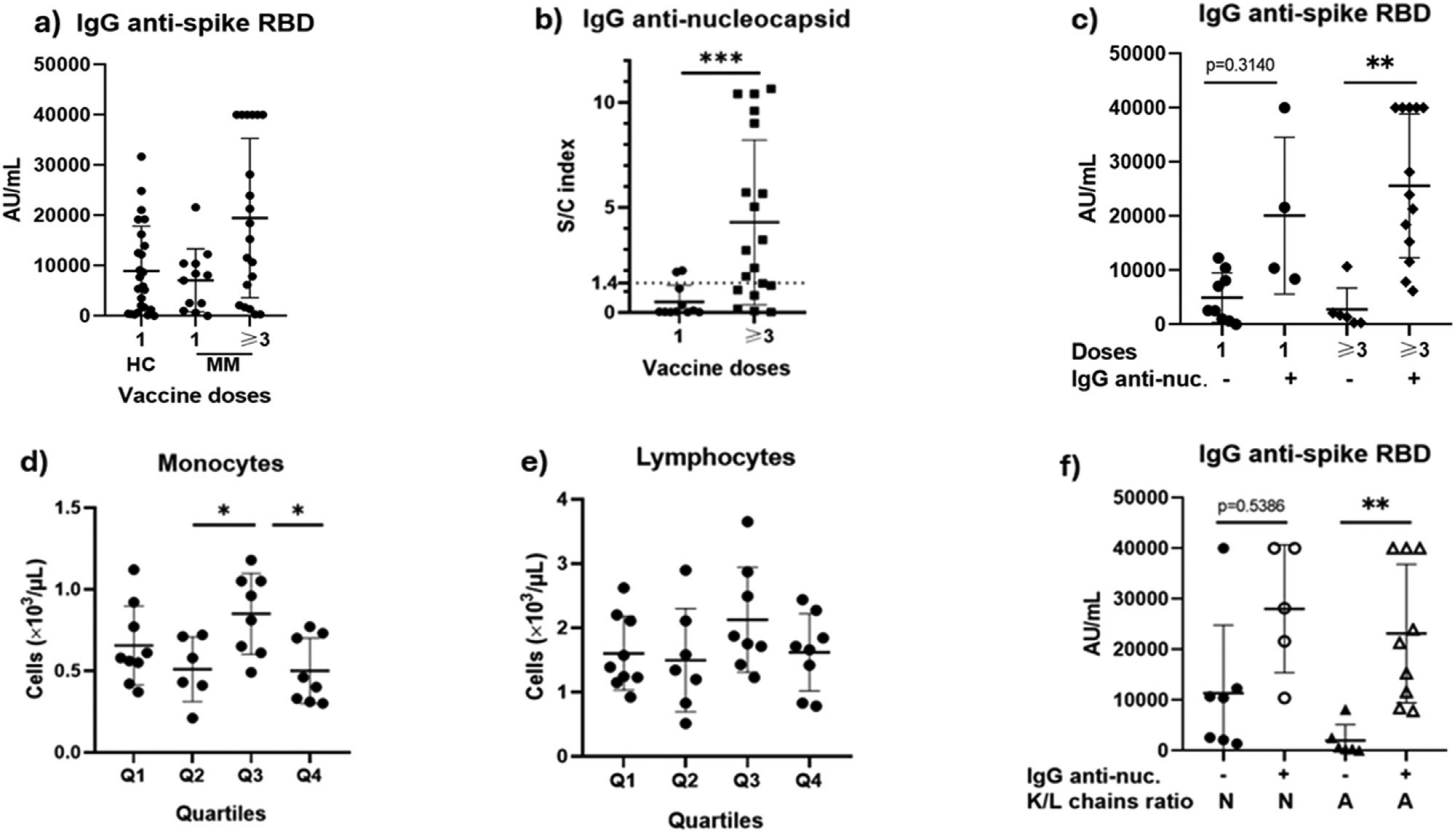
Evaluation of anti-spike receptor-binding domain (RBD) immunoglobulin G (IgG) concentrations in patients with multiple myeloma (MM). a) Serological assessment of levels of anti-spike RBD IgG in healthy controls and MM patients, and b) anti-nucleocapsid IgG of patients who received 1 dose of anti-SARS-CoV-2 vaccine and three or more doses; c) Comparison based on the vaccination doses and anti-nucleocapsid IgG positive or negative status; d) Assessment of monocytes and e) Lymphocytes in the complete blood count of peripheral blood according quartile classification of anti-spike RBD IgG antibody levels (Q1 ≤ 2523.2 AU/mL, Q2 = 2523.2–10,427.9 AU/mL, Q3 = 10,428.0–23,954.0 AU/mL, and Q4 ≥23,955.0 AU/mL); f) Analysis of patients with a subclinical infection and normal (N) or abnormal (A) kappa/lambda serum free chain ratio. The differences between the groups means were evaluated using one-way ANOVA followed by Tukey's post hoc test, or Kruskal-Wallis followed by Dunn's post hoc test for multiple comparisons. P-value <0.05 was considered statistically significant.

The IgG levels of anti-Severe Acute Respiratory Syndrome Coronavirus 2 (SARS-CoV-2) were compared between healthy controls (with a single dose of the vaccine when they were included in the study) versus MM patients (with a single dose and ≥3 doses). A significant difference was found between the study groups in the non-parametric analysis (p-value <0.05); however, in the multiple comparison analysis no differences were identified between the study groups (p-value >0.05) (Figure 1a). Additionally, the group receiving three or more doses showed significantly elevated levels of anti-nucleocapsid antibodies above the cutoff point of 1.4 S/C (p-value <0.001), thus suggesting an unreported prior subclinical infection (Figure 1b). No significant differences were observed between the groups in other laboratory findings. The patients were divided into groups based on their vaccination schemes and anti-nucleocapsid serological status. The group of MM patients that received three or more doses and with anti-nucleocapsid antibodies showed higher levels of IgG against spike RBD compared to those who had lower values of anti-nucleocapsid antibodies (p-value = 0.0024) (Figure 1c).

* P-value < 0.05, ** P-value < 0.01, and *** P-value < 0.001.

In a logistic regression model, this study aimed to predict whether high levels of Anti-spike RBD IgG were associated with the presence of anti-nucleocapsid antibodies. Although a significant relationship was found between these variables (p-value = 0.0007), the practical impact was minimal, as indicated by the odds ratio (1.000) and a small coefficient B (−0.000138). Therefore, anti-spike RBD levels are not a strong predictor of subclinical infection in this patient cohort. Additionally, non-significant correlations were observed between serum anti-spike RBD IgG levels and various laboratory parameters, including the lymphocyte count (Pearson's: *r* = 0.0310; p-value = 0.8650), monocyte count (Spearman's: *r* = −0.0930; p-value = 0.6120), total serum proteins (Pearson's: *r* = 0.1790; p-value = 0.3280), and serum gamma globulins (Spearman's: *r* = 0.1980; p-value = 0.2860).

The levels of anti-spike RBD antibodies were classified in MM patients into quartiles to assess potential differences in laboratory findings. The quartile ranges were as follows: Q1 ≤2523.2 AU/mL, Q2 = 2523.3 –10,427.9 AU/mL, Q3 = 10,428.0–23,954.0 AU/mL, and Q4 ≥23,955.0 AU/mL. MM patients with anti-spike RBD antibodies in Q3 had an elevated number of monocytes compared to Q2 (p-value <0.05) and Q4 (p-value = 0.0216) (Figure 1d). No other significant changes were observed regarding lymphocytes (Figure 1e) or other laboratory findings. Additionally, based on the normal Serum-Free Kappa/Lambda (κ/λ) light chain ratio reference range (0.26–1.65), patients were grouped to assess whether abnormal ratios were associated with significant changes in the anti-spike RBD IgG levels in patients with a subclinical infection (Figure 1f). Among patients exhibiting an abnormal κ/λ ratio, those with detectable Anti-nucleocapsid IgG demonstrated significantly higher RBD IgG titers (p-value <0.01). These data suggest that despite the presence of underlying monoclonal plasma cell proliferation, these individuals maintained a robust humoral response following subclinical SARS-CoV-2 infection. There are no significant differences in any other laboratory findings between the groups with an abnormal κ/λ ratio compared with those who had a normal ratio.

Additionally, the antibody response was evaluated in the patients who had undergone ASCT compared to those who had not. No statistically significant differences were observed in anti-spike RBD IgG levels (p-value = 0.8709) or anti-nucleoprotein antibodies (p-value = 0.1011).

## Discussion

Patients with MM often exhibit compromised immune responses due to impaired antigen presentation and antibody production. In this cohort, no significant differences were observed in anti-spike RBD IgG antibody levels between the healthy control group (individuals who had received a single vaccine dose) and MM patients who had received one or three doses. This contrasts with a previous study reporting that smoldering and symptomatic MM patients had a lower antibody response compared to healthy individuals [6]. The findings of this study suggest that, in this cohort, the protective anti-SARS-CoV-2 immune response was not significantly affected by MM treatment. It is important to mention that patients who received three or more vaccine doses tended to have higher antibody levels compared to the patients who received a single dose; this finding suggests that multiple vaccine doses may help overcome these limitations, enhancing vaccine immunogenicity. This is supported by a study showing that a third dose of the mRNA COVID-19 vaccine significantly improved the humoral response, even in individuals who did not respond to the first or second dose [5]. It is important to mention that the presence of anti-spike antibodies in serum is not an indicator of neutralizing antibodies, as up to one third of MM patients did not present virus-neutralizing antibodies, even after two doses of the vaccine, depending on their race, disease status and treatment [7].

Regardless of the inclusion criteria considered, some patients were found to have a possible subclinical infection identified through the presence of positive IgG antibodies against the viral nucleocapsid. Patients with higher levels of anti-nucleocapsid antibodies also showed elevated anti-spike antibody levels; this association was statistically significant among those with more than three vaccine doses. The elevated levels of antibodies, anti-nucleocapsid and anti-spike RBD, were also reported after six months of acute SARS-CoV-2 infection in 9/10 MM patients [8], indicating that the presence of anti-nucleocapsid antibodies in asymptomatic MM patients suggests prior exposure to SARS-CoV-2.

The present analysis revealed no significant differences in anti-spike RBD IgG or anti-nucleocapsid antibody levels between ASCT recipients and non-recipients. This finding suggests that prior ASCT may not independently impair humoral responses to COVID-19 vaccination in MM patients, at least among those with adequate immune reconstitution (e.g., vaccinated ≥6 months post-transplant). Notably, all ASCT recipients in the current cohort were on lenalidomide or bortezomib maintenance, which may have supported immune recovery [5]. However, the small sample size limits definitive conclusions, and larger studies stratifying patients by time since transplantation, conditioning regimens, and maintenance therapies are warranted.

In the context of MM, it is relevant to explore whether these patients rely on cellular immunity to compensate for their limited humoral response due to their immunocompromised status. To address this, the cellular response was analyzed depending on the levels of anti-spike RBD antibodies. This showed that patients with antibody levels in the third quartile, displayed elevated monocyte counts. The increase in monocytes within this group may indicate innate immune activation related to vaccination or a prior subclinical infection. In MM patients, monocytes might play a compensatory role in innate immunity due to deficits in adaptive responses. Alternatively, this could represent a marker of mild systemic inflammation, as the SARS-CoV-2 virus can infect monocytes via Fcγ-receptors, activating NLRP3 inflammasomes, resulting in the secretion of IL-18 and IL-1β, cell death by pyroptosis, contribute to systemic inflammation, severe COVID-19, and dissemination of the virus from the lungs [9,10]. The main limitations of this study are the small cohort size, the lack of an alternate method to measure anti-nucleocapsid IgG antibodies, and the potential for previous infection and neutralizing antibodies were not determined.

On the other hand, anti-RBD IgG levels provide valuable insights as they do not fully capture functional immunity and thus cannot be utilized as a marker of serological response [11]. Neutralizing antibodies and T-cell responses are critical for protection, especially in immunocompromised cohorts. Future work should integrate viral neutralization tests and Interferon-gamma release assays to evaluate functional immunity.

This study also reflects the reality of COVID-19 vaccination in Latin America, where heterogeneous vaccine regimens, including mixed platforms (mRNA and adenovector) and variable dosing intervals, were implemented due to supply constraints [12]. While this diversity limits direct comparisons of immunogenicity across specific vaccine types, it enhances the generalizability of our findings to real-world clinical practice in resource-variable settings. Notably, the data of this study suggest that the number of doses had a greater impact on anti-spike IgG levels than the vaccine platform itself. However, unmeasured confounding variables (e.g., interval between doses and prior undiagnosed infections) may influence these results. Future studies in similar contexts should stratify analyzes by both vaccine type and dosing intervals to better isolate these effects.

## Conclusion

Similar Anti-spike RBD IgG levels were found in patients with MM compared to controls and patients with three doses of anti-SARS-CoV-2 vaccine showed higher anti-spike RBD antibodies, suggesting the treatment did not affect the antibody response in these patients.

## Clinical practice points

• Prioritize ≥3 COVID-19 vaccine doses for MM patients to enhance antibody responses.
• Monitor for subclinical SARS-CoV-2 infections via anti-nucleocapsid antibody testing in vaccinated MM cohorts.
• Consider innate immune markers (e.g., monocytes) as potential indicators of vaccine-related immune activation.
• Reassess vaccination schedules in MM patients on immunomodulatory therapies to optimize long-term protection.

## Conflicts of interest

The author declares no conflicts of interest.
